# Do plant communities show constant final yield?

**DOI:** 10.1002/ecy.3802

**Published:** 2022-08-18

**Authors:** Andrea Cavalieri, Dorothee Groß, Alexandra Dutay, Jacob Weiner

**Affiliations:** ^1^ Department of Plant and Environmental Sciences University of Copenhagen Frederiksberg Denmark; ^2^ Institute of Landscape and Plant Ecology University of Hohenheim Stuttgart Germany; ^3^ FNSEA, Centre Val de Loire Orléans France

**Keywords:** biomass production, community regulation, density dependence, plant populations

## Abstract

Total biomass production of plant monocultures growing over a range of densities and harvested after a period of growth increases monotonically with density and then levels out at higher densities. This pattern is called constant final yield (CFY) and is considered one of the most general phenomena in plant ecology. If CFY applies to plant communities, it would be a key to understanding and predicting many community‐level phenomena. We tested two primary hypotheses experimentally: (1) Mixtures of several species show CFY. (2) If so, the proportion of biomass production by the component species in a mixture does not change at densities above the density that reaches CFY. We performed a series of glasshouse experiments over 3 years using a “community density series,” in which the overall density of five species was varied while their proportions remained unchanged. In the first experiment, we grew a mixture of annual and perennial herbaceous species in mesocosms, and all species were also grown in monocultures at the corresponding densities. A similar experiment was performed in the second and third years, but only with annuals. A third experiment with mixtures only was performed in pots over 2 years. In all cases, aboveground biomass was harvested, separated by species, dried, and weighed. Perennials with underground storage organs produced maximum aboveground biomass at low or intermediate densities. In the second experiment, two of the species produced maximum biomass at the second‐highest density in monoculture, while mixtures of all five species showed classical CFY behavior, and the contribution of the species to the mixture changed very little above the lowest density producing CFY. The results of the third experiment were also consistent with the hypotheses. In conclusion, CFY in aboveground biomass production was observed in communities of annual species, and the contribution of the individual species was relatively insensitive to an increase in density above that reaching CFY, i.e., competitive performance of the species changed with density until CFY was reached. Evidence for CFY was stronger in mixture than in monoculture. Coexistence theory must include density as well as frequency dependence if densities are below CFY.

## INTRODUCTION

When single‐species plant stands are grown over a wide range of initial densities and harvested after a period of growth, total biomass production initially increases linearly with density at very low densities, then levels off, and then remains constant at higher densities. This pattern is called constant final yield (CFY) and is considered one of the most robust and compelling patterns in plant population ecology (Silvertown & Charlesworth, 2001). If CFY applies to plant communities as well as single‐species populations, it would be a key to understanding and predicting many community‐level phenomena, providing a major constraint on plant community behavior and a much‐needed operational definition and measure of disturbance (Weiner & Freckleton, 2010). Populations grow until they are limited by competition for resources (Darwin, 1869; Tilman, 1982), and intense competition is observed in most of the world's natural and cultural plant communities. Unless all species are competitively equal, as in “neutral” theory (Hubbell, 2001), competition will be an important driver of community dynamics. The widespread occurrence of extensive density‐dependent mortality (self‐thinning), which requires that populations be at CFY (Weiner & Freckleton, 2010), suggests that many, although by no means all, natural and seminatural plant communities will be at or near CFY.

The essential prediction of CFY is that initial biomass production increases with density and levels off, while not decreasing at ever‐higher densities. Considering the whole biomass versus density curve in a density series experiment, there are two types of results that would be evidence against CFY: (1) Biomass production levels off at high densities, but it is higher at some lower density. (2) Biomass production decreases from high to higher densities. According to the only attempt at a comprehensive review of which we are aware (Wille, 2012), the results of most experiments on single‐species populations that have been grown over a wide enough range of densities to address this are consistent with CFY, although there are exceptions. We do not have appropriate data to ask whether CFY occurs at the community level. We might expect that a mixture of several species, each of which shows CFY in monoculture, would also show CFY, but changes in the competitive performance (CP) of species at different densities or nonlinear interactions among species could result in reduced biomass production in stands consisting of many species at high densities, even if each species shows CFY in pure stands. Here we report on experiments designed to address this question.

If CFY does occur at the community level, as we hypothesize, does density have an effect on the community composition among densities producing CFY? We know of only one study relevant to this question (Stachová et al., 2013), and its results were not consistent. Data on two‐species mixtures grown at different densities in “replacement series” experiments (Taylor & Aarssen, 1989) showed no change in the biomass production of two species among densities producing CFY if their initial proportions were the same at all densities. If this result can be generalized, it would mean that CFY sets a very clear limit on the behavior of crowded plant communities, which may apply to the many natural and cultural plant communities that are at CFY. This is important because it would support the idea of “community regulation” in the same sense as population regulation, i.e., within certain limits resulting from stochastic factors and given a species pool, plant communities will converge in composition despite differences in initial composition. Though studies varying species proportions are necessary to provide strong support for such a theory (e.g., Hart et al., 2016; Petry et al., 2018; Turnbull et al., 2013), such studies are insufficient if they are not supported by studies of the effects of density (Damgaard & Weiner, 2008; Stachová et al., 2013) because density may change the effect of proportions, and variation in density over time is ubiquitous in plant communities.

The hypothesis that the outcome of competition at the community level is not highly sensitive to changes in total density above a natural limit, which is often reached in the field, is an essential assumption if communities are to be self‐regulating. If community dynamics are highly sensitive to the fluctuations in density observed in nature, plant community behavior will be very difficult if not impossible to predict. Low density produces “transient dynamics” of populations not constrained by density dependence (Foster & Tilman, 2003). Thus, investigations of both species' proportions and density in plant communities are necessary if we are to test the hypothesis of community regulation.

In short, if the amount of biomass produced by different species established in specific proportions at different densities does not vary among those densities producing CFY, this would support theories of community regulation and convergence. Despite the many plant density experiments that have been performed over recent decades, this central question of CFY of plant communities has not been addressed. First, because most experiments have been performed in specific applied contexts (e.g., agriculture), so the range of densities investigated is very limited from an ecological perspective, with no or only one density high enough to produce CFY. Second, many studies measure only specific yield components (e.g., harvestable yield), not biomass production.

Biomass production is a central functional activity of plant communities. Though ecologists have learned that there is no single “ultimate” currency in ecology, biomass is one of the central currencies for plants, because it is closely related to energy, i.e., net primary production. Since plants are primarily made of carbohydrates, biomass is very highly correlated with energy content (Hickman & Pitelka, 1975), and most measurements of net primary production are based on biomass. In addition, plant growth is modular. Plants grow primarily by producing more, not bigger, modules. Most biodiversity–productivity experiments to date have not paid sufficient attention to the role of density. Several studies have shown that some plant community behaviors that have been attributed to biodiversity are actually attributable to density, which varied among the diversity treatments (He et al., 2005; Stachová et al., 2013). These studies concluded, as Weiner and Freckleton (2010) did, that we must investigate whether CFY occurs at the community level. If it does, then biodiversity experiments simply need to be performed at high enough densities if the communities being investigated are at CFY.

In summary, corroboration of the hypothesis of community CFY will have important implications for both basic and applied plant ecology and enable the development of more robust predictive models of the dynamics of plant communities and better assess the role of disturbance in community dynamics.

We test the following hypotheses:

1. Primary hypotheses

Hypothesis 1 *CFY occurs at the community level, i.e., in a mixture of plant species grown at specific initial proportions at a wide range of densities, biomass production does not decrease with density at high densities.*

Hypothesis 2 *The biomass contribution of each species to community CFY growing in specific proportions does not change with increasing community density above the lowest density producing CFY.*

2 Secondary hypothesis

Hypothesis 3 *(a) Total biomass production by a plant community at CFY can be predicted from the biomasses of the individual species at CFY in monoculture and their proportions in the mixture. Alternative hypotheses are that (b) the CFY of a community reflects the behavior of the species that produces the largest biomass in monoculture and (c) overyielding in mixtures makes the prediction of community biomass from monocultures impossible.*

## MATERIALS AND METHODS

The simplest and most direct way to address CFY of plant communities is to use what's known as a community density series (Goldberg et al., 1995), in which the overall density of several species growing together is varied while their proportions remain unchanged. Three experiments were conducted during the spring–summer seasons of 2016–2018 (Table 1). The first and second experiments were conducted in mesocosms (large containers) in a greenhouse at an experimental farm of the University of Copenhagen, in Taastrup (55°40′07.2″ N, 12°18′19.8″ E), and the third in pots in a greenhouse of the University of Copenhagen in Frederiksberg (55°41′08.5″ N, 12°32′38.2″ E).

**TABLE 1:** Diagram summarizing number of years, size of container, seed density, and number of replicates conducted per each experiment.

Experiments	Year	Container	Density	Mixture	Monoculture
Experiment 1	2016	Mesocosm 48 L	g seeds m^−2^	3 replicates	3 replicates
Experiment 2	2017	Mesocosm 48 L	g seeds m^−2^	3 replicates	1 replicate
	2018	Mesocosm 48 L	g seeds m^−2^	3 replicates	1 replicate
Experiment 3	2017	Pot 20 L	No. seeds m^−2^	3 replicates	…
	2018	Pot 20 L	No. seeds m^−2^	3 replicates	…

### Plant species

Because of difficulties in controlling and predicting germination of seeds collected in the field, we used “model” weed communities of five species of minimally domesticated horticultural plants that are closely related, and in some cases identical, to naturally occurring weeds, but were screened and bred for high germination rates. The species selected for the first experiment (2016) were *Malva sylvestris* L. (high mallow), *Plantago lanceolata* L. (narrowleaf plantain), *Rumex acetosa* L. (garden sorrel), *Lolium multiflorum* Lam. (Italian ryegrass), and *Trifolium repens* L. (white clover). Based on the results of the first experiment, we decided to restrict the communities to annual or annual‐like species, so in the second mesocosm experiment, repeated over 2 years (2017 and 2018), *M. sylvestris*, *P. lanceolata*, and *R. acetosa* were replaced with *Dracocephalum moldavica* L. (Moldavian dragonhead), *Centaurea cyanus* L. (cornflower), and *Plantago psyllium* L. (psyllium); *T. repens*, which behaves as an annual under controlled conditions (Hutchinson et al., 1995) and does not have a large underground storage organ, was retained. For the pot experiment, also repeated over 2 years, the plant species studied were the same as in Experiment 2, except that *T. repens* was replaced with *Calendula officinalis* L. (English marigold). Commercial seeds were purchased from Pharmasaat Arznei‐ und Gewürzpflanzensaatzucht GmbH (Artern/Unstrut, Germany) and Sow Seeds Limited (Brough, UK).

### Sowing densities

Before starting the experiment, germination tests were performed in 10‐cm petri dishes, and target densities for sowing were calculated based on the germination rate. The experiments conducted in the two types of containers differed in the method used to calculate sowing densities; in Experiments 1 and 2 (mesocosms), density was based on seed weight (g seeds m^−2^), whereas in Experiment 3 (pots) density was based on seed number (number seeds m^−2^). Since the species vary greatly in seed mass, in the experiment using seed weight, we sowed the species in equal proportions in total seed mass, not seed number, at each density, starting with the lowest density of 12 seeds m^−2^ for the species with the largest seeds (*M. sylvestris*, thousand‐seed weight = 7.98 g) at the lowest density. The density series was logarithmic, corresponding to densities of 12, 60, 300, 1500, and 7500 seeds m^−2^ of *M. sylvestris*, henceforth referred to as Densities 1, 2, 3, 4, and 5, respectively. For each density, seeds of *M. sylvestris* were counted and weighed, and this weight was then used for the other species. To obtain more information about the behavior of the species at high densities, the target seed density in Experiment 2 and 3 was increased to 50, 175, 610, 2140, and 7500 seeds m^−2^. In Experiment 2, the species with the largest seeds was *C. cyanus* (thousand‐seed weight = 3.98 g). The density series was based on densities of 50, 175, 612, 2140, and 7500 seeds m^−2^ of *C. cyanus* (Densities 1–5, respectively). Thus, at each density in the experiments based on seed mass, the number of seeds differed among species, but the proportions were the same. In the pot experiment based on seed number, seeds of each species and for each density were counted, adjusted to the real germination rates based on a germination test performed on petri dishes, and the sown at equal predicted emergence density for each overall density. Populations were grown in monoculture and in a five‐species mixture (mixed cultures) in Experiments 1 and 2 and, owing to space and logistical constraints, only in mixtures in Experiment 3.

Fruits produced were collected throughout the growing season. At the end of the experiments, all aboveground biomass was cut at the soil surface, sorted by species, and further divided into vegetative biomass and fruits/seeds, which, together with any earlier fruits produced, constituted total plant biomass. This was used to determine CP, sometimes referred to as competitive ability, as defined by Stoll and Prati (2001): (biomass in mixture × 5)/(biomass in monoculture). CP was calculated for each plant species at each density in Experiments 1 and 2. We could only investigate the effect of density on survival (self‐thinning) in Experiment 3 because in this experiment a specific number of seeds of each species were sown, so counting the number of surviving plants at the end of the experiments gave us a measure of survivorship. To test the null hypothesis that the total biomass production by a plant community at CFY can be predicted from that of the individual species and their proportions, we calculated a “null” predicted total biomass: the sum of one fifth of the total green biomass produced by each of the individual five species when growing in monoculture for Experiments 1 and 2.

### Mesocosm experiments—Experiments 1 and 2

Mesocosms (48‐L containers, 45 cm Ø, 30 cm height) were used to correspond as well as possible to field conditions. Soil was collected from the field of the experimental farm. It is a sandy clay (pH 6.9 [CaCl_2_], 12.8 mg P kg^−1^ soil, 4.7 mg K kg^−1^ soil, 2.2 mg kg^−1^). Before the containers were filled, the soil was sieved, and commercial potting soil (SW Horto A/S, Bramming, DK) was added (10% of volume) as a source of nutrients (pH 5.5–6.5, NPK 14‐7‐15) and organic matter. The soil was then homogenized using a soil mixer. After being filled with soil, the pots were watered for 2 weeks to favor the germination of potential weed seeds in the natural soil seedbank, and weeds that emerged were hand weeded. Seeds were hand sown by spreading evenly over the soil surface.

In Experiment 1, the five plant species were sown in monocultures and in a five‐species mixture at five different densities in a community density series. Experiment 2, performed in the second and third years, three of the species were changed as described previously and, due to space and logistical constraints, there was only one replicate of each monoculture. The mesocosms were arranged on wood pallets placed on the floor in a randomized complete block design with three replicates. Sowing was performed in May of each year (Experiment 1 on Julian Day 133 in 2016 and Experiment 2 on Julian Days 125 and 122 in 2017 and 2018, respectively). The seeds were then lightly covered with the soil mix, and the soil was watered afterwards. The final harvest was performed when many of the plants had reached maturity (Julian Day 201 for Experiment 1 and Days 185 and 177 in 2017 and 2018, respectively, for Experiment 2).

### Pot experiment—Experiment 3

The experiment was conducted in plastic pots (20‐L containers, 32 cm Ø, 27 cm height). Potting soil (the same as used as a supplement in Experiment 2) and Perlite were mixed at a 75:25 ratio. Sowing was performed in April (Julian Days 97 and 106 in 2017 and 2018, respectively), and then the soil was watered. The pots were placed on the floor and arranged in a randomized complete block design with three replicates. The experiment was conducted with a mixture of five plant species at five densities, but no monoculture experiment could be conducted. Because a specific number of seeds of each species were sown in this experiment, the effect of density on survival (density‐dependent mortality) was calculated as the difference in the number of plants between seedling emergence and harvest date in both years: Percentage survival = [(number of plants harvested)/(number of plants emerged)] × 100.

For all the experiments, air temperature was maintained at 15–20°C, corresponding to the natural summer conditions of the region. When temperatures in the greenhouses were too high, a curtain was automatically activated, but no cooling system was available. No fertilizer was added during the experiments, nor was supplemental lighting supplied. Local tap water was used for irrigation, and pests and diseases were treated as needed. The final harvest was conducted in June (Julian Days 157 and 164 in 2017 and 2018, respectively). Individual plants of each species were counted as they were being harvested.

### Statistical analyses

Analyses were performed using mixed‐effect ANOVA models with plant species and density as fixed effects and replicate blocks and pot position as a random effect. We are not modeling density effects here because the effects are expected to be curvilinear in a way that cannot be easily linearized or modeled simply, so density is treated as a qualitative variable but can be clearly observed graphically. All data were tested for homogeneity of residuals. Data were analyzed using SAS 9.4 with PROC MIXED (SAS Institute Inc., 2015).

## RESULTS

### Experiment 1

**FIGURE 1:**
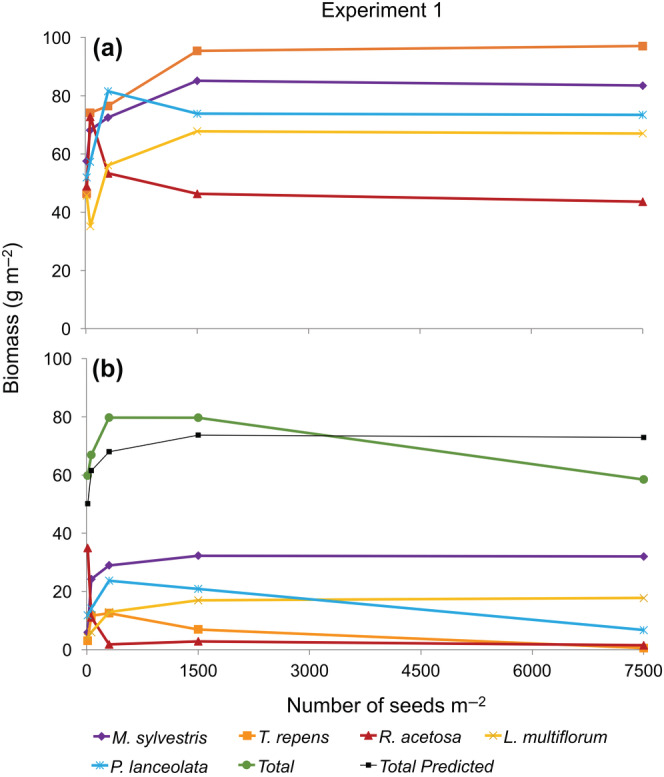
Biomass–density relationships in (a) pure and (b) five‐mixture species for Experiment 1 in mesocosms in 2016. Total predicted is based on monocultures.

Biomass production of the monocultures was affected by species and density, but there was no significant interaction (Table 2). The highest plant biomass production was observed at the two highest densities for *M. sylvestris*, *T. repens*, and *L. multiflorum*, whereas for *R. acetosa* and *P. lanceolata* the highest biomass production occurred at the second and third densities, respectively (Figure 1a). In monoculture, the biomass production of all the species did not differ between the two highest densities. *T. repens* had the highest biomass production in monoculture, followed by *M. sylvestris*, *P. lanceolata*, *L. multiflorum*, and *R. acetosa* (Figure 1a).

**TABLE 2:** Statistical results of PROC MIXED model used to test species and density effect in experiments conducted in monocultures (Experiments 1 and 2) and mixtures (all three experiments).

Experiment	Year	Stand	Factor	Variable	Num DF	Den DF	*F*	*p* value
1	2016	Mono	Species	SpBio	4	48	7.32	0.0001
			Density		4	48	3.98	0.0072
			Species × density		16	48	1.54	0.1252
		Mix	Density	ComBio	4	10	74.86	<0.0001
			Species	SpBio	4	50	4.91	0.0020
			Density		4	50	49.56	<0.0001
			Species × density		16	50	21.13	<0.0001
2	2017	Mono	Species	SpBio	4	16	5.52	0.0055
			Density		4	16	16.07	<0.0001
		Mix	Density	ComBio	4	10	39.63	<0.0001
			Species	SpBio	4	50	163.22	<0.0001
			Density		4	50	20.05	<0.0001
			Species × density		16	50	5.24	<0.0001
	2018	Mono	Species	SpBio	4	16	5.16	0.0073
			Density		4	16	29.96	<0.0001
		Mix	Density	ComBio	4	10	17.30	0.0002
			Species	SpBio	4	50	156.62	<0.0001
			Density		4	50	17.00	<0.0001
			Species × density		16	50	5.31	<0.0001
3	2017	Mix	Density	ComBio	4	10	10.42	0.0014
			Species	SpBio	4	50	195.02	<0.0001
			Density		4	50	6.91	0.0002
			Species × density		16	50	9.02	<0.0001
	2018	Mix	Density	ComBio	4	8	2.72	0.1066
			Species	SpBio	4	48	181.77	<0.0001
			Density		4	48	2.99	0.0278
			Species × density		16	48	10.30	<0.0001

*Note*: Stand types and years were analyzed separately.

Abbreviations: ComBio, total community biomass of mixture; Mix, five‐species mixture; Mono, monoculture; SpBio, biomass of a species in mixture or monoculture.

In the five‐species mixture, total community biomass was affected by density, whereas the biomass contribution of each species was affected by species, density, and their interactions (Figure 1b, Table 2). Looking at the contribution of individual species to this total, only *M. sylvestris*, the most competitive species at all but the lowest densities, and *L. multiflorum* showed classical CFY behavior within the mixture, increasing with density and then leveling off at the higher densities. The biomass of *P. lanceolata* and *T. repens* was highest at the middle density, whereas for *R. acetosa* biomass decreased with density and remained extremely low over the three highest densities. The decrease in the total biomass of the mixture from the fourth to the highest density was due to the decrease in *P. lanceolata* and, to a much lesser extent, *T. repens*. *T. repens* produced the largest amount of biomass at the two highest densities in monoculture but was the second most suppressed species in mixture. Seed production in the five‐species mixture occurred only in *L. multiflorum*, *M. sylvestris*, and *P. lanceolata* (Table 2; Appendix S1: Table S3).

The null model, based on monoculture production, predicted a lower total biomass production than observed in real mixtures, in a typical CFY pattern, increasing with density and leveling off at the two highest densities (Figure 1b).

### Experiment 2

**FIGURE 2:**
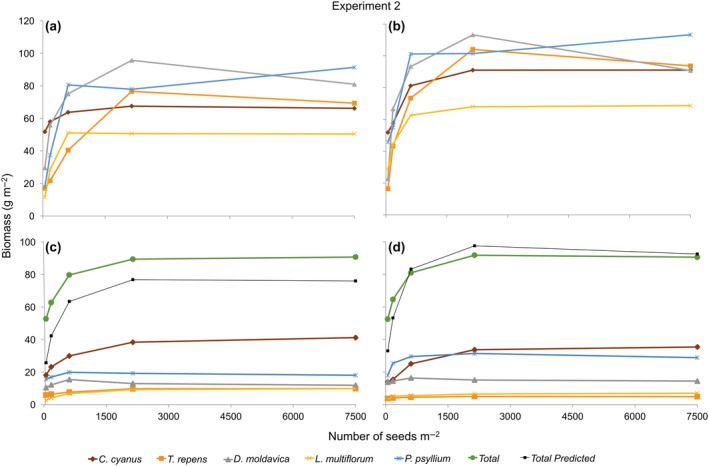
Biomass–density relationships in (a, b) pure and (c, d) five‐mixture species, respectively, for Experiment 2 in 2017 and 2018. Total predicted is based on monocultures.

Biomass produced in the monoculture stands in Experiment 2 was affected by density in both years (Table 2). Two species (*C. cyanus* and *L. multiflorum*) clearly showed CFY behavior, two species (*P. psyllium* and *T. repens*) exhibited behavior very similar to CFY, and one species (*D. moldavica*) showed a decline in biomass production from the next‐highest to the highest density (Figure 2a,b). In both years the highest biomass production was observed for *D. moldavica* and *P. psyllium* at the fourth and fifth densities, respectively (Figure 2a,b). *T. repens* showed the third highest biomass production overall, with a peak at Density 4, whereas *L. multiflorum* and *C. cyanus* had the lowest and second lowest biomass, respectively.

The total community biomass of the five‐species mixture was affected by density in both years (Table 2) and showed the classical CFY pattern, with an initial increase from Density 1 to Density 4, then leveling off from Density 4 to 5 (Figure 2c,d). The contribution that each species made to the total biomass production differed slightly between the 2 years, although the ranking was the same in both years. All species showed classical CFY behavior when growing in mixture. The biomass of two of the species, *T. repens* and *L. multiflorum*, changed very little with density above the lowest density.

The predicted community biomass based on the monocultures showed a typical CFY pattern in both years, with an increasing biomass production up to Density 4, leveling off from Density 4 to 5 (Figure 2c,d). The total biomass of the mixture predicted by our null model was lower than observed in the mixture in 2017, but the model predicted mixture production quite well in 2018. The proportion of biomass of each species in a mixture did not reflect monoculture biomass production, however.

### Experiment 3

**FIGURE 3:**
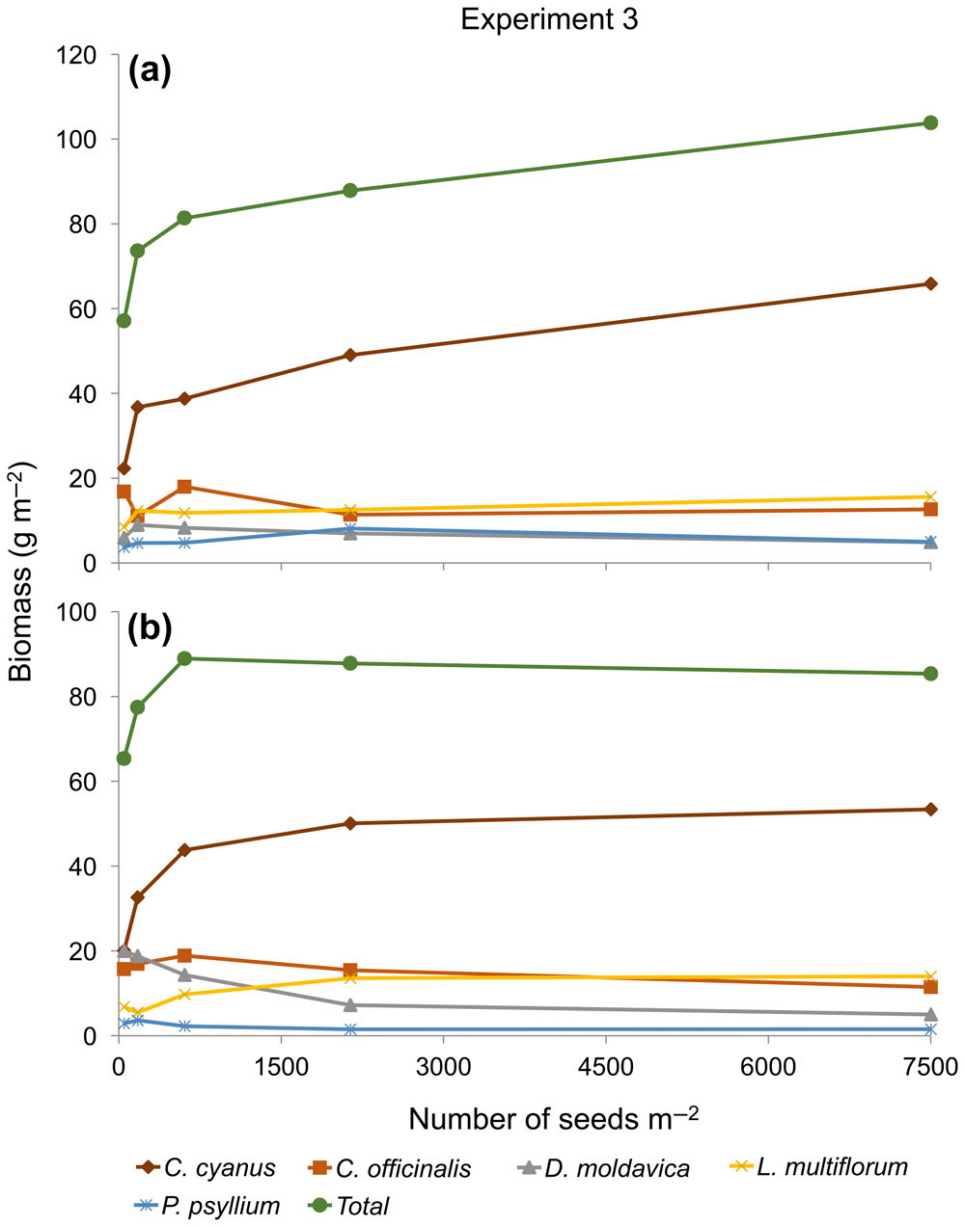
Biomass–density relationships for five‐species mixtures in Experiment 3 in (a) 2017 and (b) 2018.

Community biomass produced in the five‐species mixture was significantly affected by density in 2017, but not in 2018 (Table 2). In 2017, community biomass production did not appear to reach CFY but increased monotonically with increasing density (Figure 3a). In 2018, community biomass was not significantly affected by density but showed CFY behavior, with the lowest density already close to CFY (Figure 3b). The contribution of each species to the total plant biomass was affected by species, density, and their interaction (Table 2).

Looking at the behavior of each species in the mixture, biomass production by all species did not differ between the two highest densities, except for *C. cyanus* in 2017, which continued to increase monotonically with density. *C. officinalis* biomass was not affected by density during 2017, whereas in 2018 its biomass production decreased slightly from a maximum at the middle density. *D. moldavica* biomass was negatively affected by density and showed the lowest biomass production at the highest density. *L. multiflorum* behaved consistently in both years, showing little variation in biomass production at the different densities. *P. psyllium* was hardly affected by density at all in both years.

### Competitive performance—Experiments 1 and 2

**FIGURE 4:**
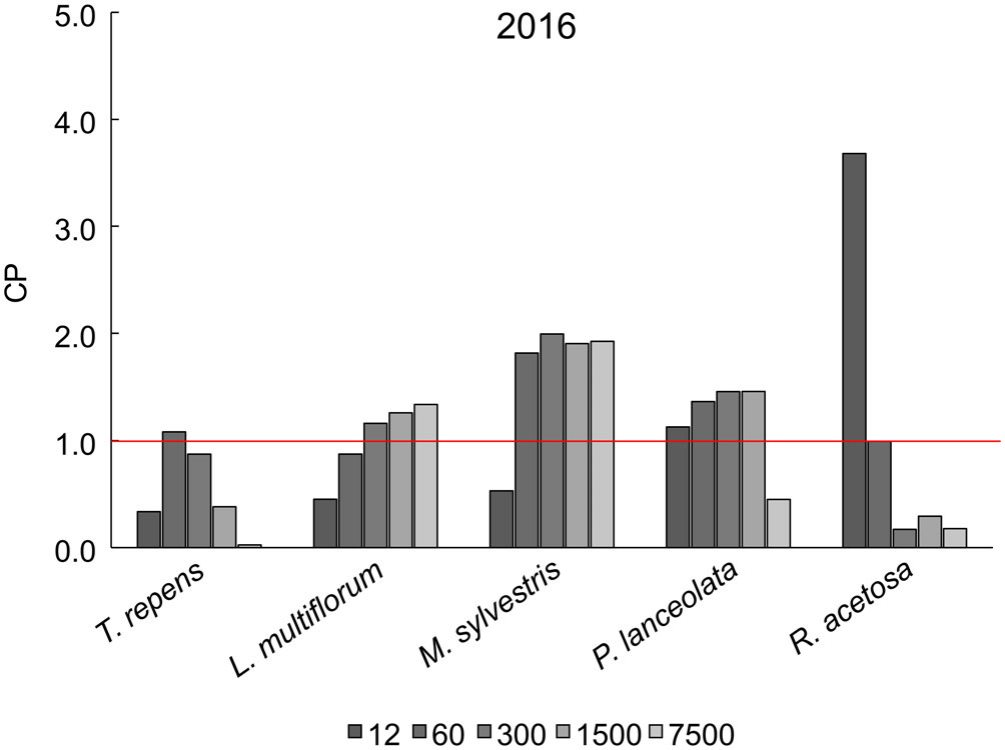
Competitive performance = (biomass in mixture × 5)/(biomass in monoculture) of the five species at the five densities in Experiment 1.

**FIGURE 5:**
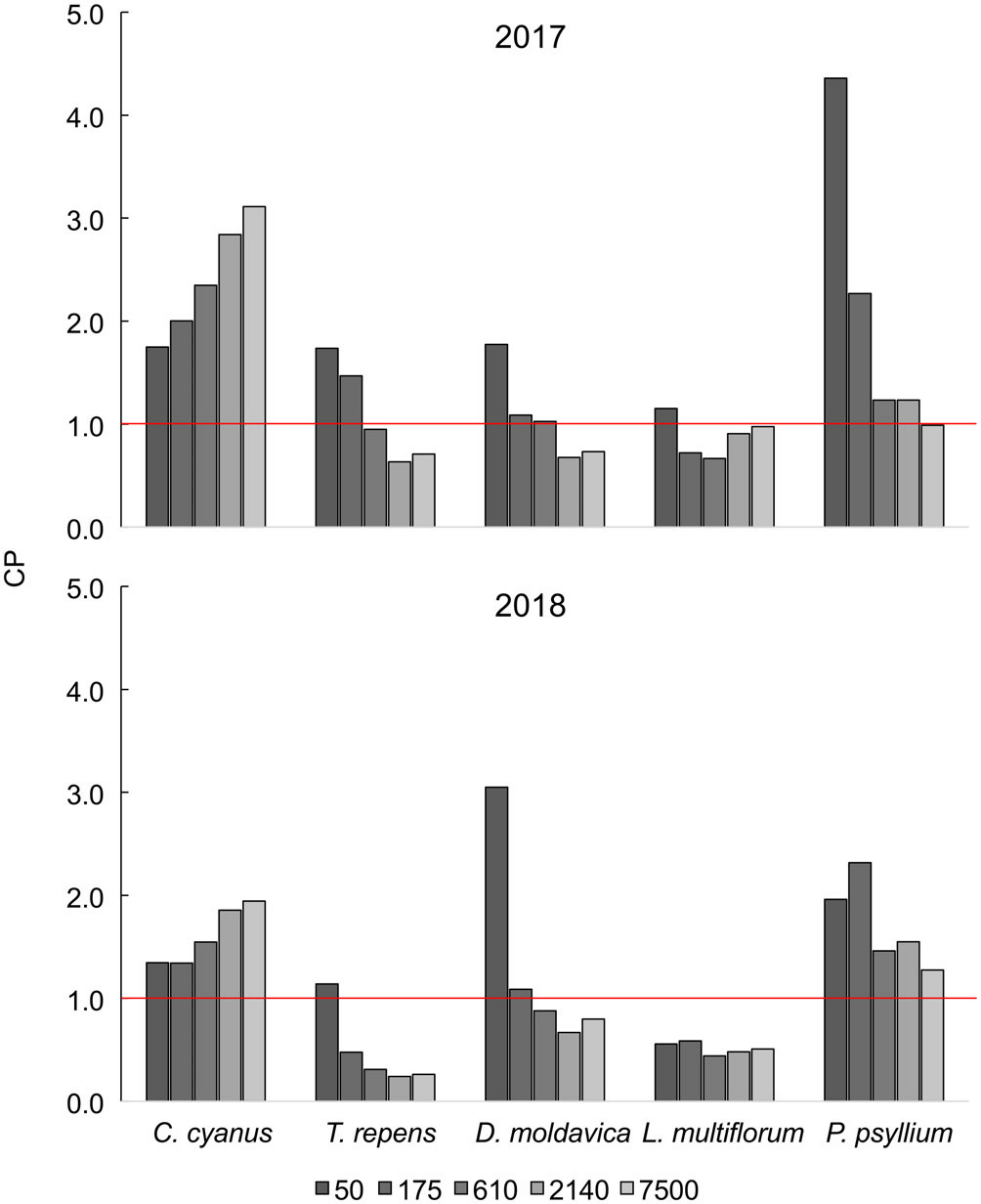
Competitive performance = (biomass in mixture × 5)/(biomass in monoculture) of the five species at the five densities in Experiment 2.

The CP of the species changed with density and, to a much lesser extent, between the 2 years in Experiment 2. In Experiment 1, *M. sylvestris* was the most competitive at all densities except the lowest, where *R. acetosa* dominated (Figure 4). *T. repens* was the weakest competitor on average across all densities but performed well at Density 2. In Experiment 2, *C. cyanus* and *P. psyllium* were generally the most competitive species, with CPs consistently higher than 1, whereas *T. repens* was the least competitive, except at low density (Figure 5a,b). *L. multiflorum* and *D. moldavica* also had high CPs at lower densities.

### Self‐thinning—Experiment 3

In several cases the number of individuals at harvest was higher than the number counted at emergence, presumably because some seeds germinated over the course of the experiment and difficulties in counting small seedlings at high density (Appendix S1: Table S11). Therefore, we are probably underestimating the amount of mortality in many cases. Significant decreases in survival were observed at the higher densities.

## DISCUSSION

### Hypothesis 1

The data observed for the five‐species mixtures were consistent with the hypothesis of CFY at the community level in three out of the five experiment‐years (both years of Experiment 2 and Year 2 of Experiment 3), inconsistent in one case (Experiment 1), and equivocal in one case (Year 1 of Experiment 3). In Experiment 1 (with annual and biennial species), community biomass production decreased at the highest density, rejecting the hypothesis of CFY in this case. In the first year of Experiment 3, the total biomass produced at the community level and by the dominant species increased monotonically at the higher densities, so this cannot be considered evidence for or against the hypothesis, because we cannot conclude that maximum biomass had been reached.

The lack of support for the hypothesis in Experiment 1 can be understood by looking at the contribution of each of the species when growing in mixture. Biomass production at the community level was strongly affected by the dominant species. The decline in biomass from the second highest to the highest densities in Experiment 1 was due to *P. lanceolata* and, to a much lesser extent, by *T. repens*, both of which showed maximum biomass at the middle density in the mixture. The same result was observed by Stachová et al. (2013), who showed that the yield of *P. lanceolata* decreases with density when growing in mixture. *P. lanceolata* is a rosette‐forming biennial with a belowground storage organ. *P. lanceolata* and the other rosette‐forming perennial, *R. acetosa*, did not show CFY in monoculture, suggesting that CFY for monocultures may not be as general as often assumed. To ask if biennials and perennials show CFY would require that all the species be close to their equilibria in a community. This could only be addressed in a long‐term experiment or at least growth over two consecutive years. For this reason, we investigated only annual plants in the later experiments.

We could not measure belowground biomass in our experiments because it was not possible to remove roots and separate the species in the five‐species mixture at the higher densities. Using aboveground biomass as a proxy for total biomass may be reasonable for wild plants in general (Postma et al., 2021), but it may be problematic for biennial or perennial plants with belowground storage organs, which is why we used only annuals in experiments after the first year. The effects of plant density on the root:shoot ratio are not consistent across different studies and appear to be highly influenced by other factors. Density increased the root:shoot ratio of *P. lanceolata* at low soil nitrogen levels, but not when nitrogen was added (Berendse & Möller, 2009), which could help explain our anomalous results with this species. Competition above ground did not affect the root:shoot ratio in *Abutilon theophrasti* plants (Casper et al., 1998). A recent review (Postma et al., 2021) found an increase in the fraction of total plant biomass in roots at higher densities in agricultural plants, but not wild plants, consistent with the assumption that the root:shoot ratio did not change much in Experiments 2 and 3 and, therefore, that aboveground biomass does reflect total biomass in these experiments.

There were relatively more clear exceptions to CFY among monocultures (six out of 15 cases) than in mixtures (one out of five cases). Niche differences may promote CFY in mixtures because resources are more efficiently used. Similarly, the effects of allometry/growth form, which can lead to decreased biomass production at very high densities in some species, are compensated by other species. CFY may be more general for communities than for monocultures.

### Hypothesis 2

Our second hypothesis, that in mixtures showing CFY the contribution of individual species to total community biomass is relatively insensitive to overall density among densities producing CFY, also received support in the three cases that showed CFY at the community level (Figures 2c,d and 3b). Changes in the contribution of the individual species to the mixtures varied very little from the second highest to the highest density. This supports our hypothesis that the competitive advantage of species in a mixture changes with density until CFY is reached but does not change much more at even higher densities. This has important implications for the coexistence of species at high densities. Size‐asymmetric competition increases with density, so we might expect that species that are suppressed at high density will be even more suppressed at even higher densities, but this was not the case. Of course, the behavior of species in specific proportions at different densities does not map directly onto the behavior of populations over generations, but our results provide evidence that less competitive species can “hold their own” in the short run in the face of competition from more competitive species at very high densities.

The CPs provide clear evidence of a tradeoff between inter‐ and intraspecific competition among the species across the densities. Intraspecific competition often has a larger negative effect in the competitively strongest species than in competitively weak species (Wassmuth et al., 2009). Density had a strong effect on CP: The CP of *C. cyanus* increased with increasing density, whereas the CP of *T. repens* decreased. Thus, our results suggest that reductions in biomass due to disturbance may benefit weaker competitors in a way somewhat different than in classical *r‐K* theory: Species that do not compete well at high density not only grow faster at low density but also compete better at low density (Figures 4 and 5). The density dependence of CP is evidence against the idea that competitive ability is general and invariant, as proposed by Grime (2006) and others.

### Hypothesis 3

Our third hypothesis, that the biomass production of individual species growing in monocultures can predict the biomass of mixtures of the species, was supported in one of the two relevant cases (both in Experiment 2), but not in the way our simple null model would predict: The combined proportional contribution of the species based on their monocultures did predict community biomass in Year 2, but it did not reflect their contributions to the mixture. *D. moldavica* was one of the most productive species in monoculture, but it was suppressed in the mixture, whereas *C. cyanus* was one of the two least productive species in monoculture at high densities, but it was the most productive in the mixture. Thus, biomass production by the individual species at high density contains information relevant to the total biomass of a mixture of the species, but not their contributions to the mixture.

There was overyielding in mixtures in Year 1 of Experiment 2, so the total biomass production of the community was underestimated by monoculture production. Empirical evidence of overyielding in species mixtures compared with monocultures has been accumulating in recent years (e.g., Hendriks et al., 2013, 2015), although it is by no means universal. The widely accepted explanation is that increased biodiversity at the mixture level contributed to increasing functional richness and niche differences, resulting in increased resource use, which is one of the two mechanisms leading to the well‐documented relationship between diversity and productivity (Lambers et al., 2004). It is important to note that overyielding in mixtures is not incompatible with CFY at the community level, as the first year of Experiment 2 demonstrated (Figure 5). It is not clear why overyielding was observed in one year but not the other, even though the experimental conditions were as similar as possible.

### Implications for plant community ecology and limitations

The implications of experiments lasting one growing season for long‐term community dynamics are, of course, limited. The fact that we could not measure belowground biomass is another limitation of our results, although this may only be important for the one experiment that included two biennial species with belowground storage organs and, probably therefore, did not show CFY for aboveground biomass production. Still, our experiments provide useful information, which can inform the design and interpretation of longer‐term studies.

Theoretical (Chesson, 2000) and empirical (Damgaard & Weiner, 2021; Levine et al., 2008) studies have emphasized the role of negative frequency dependence resulting from niche differences for the long‐term coexistence of plant species, but the role of density in this should not be underestimated. Frequency dependence will change with density and may even shift from positive to negative. Temporal variation in density is ubiquitous in plant communities due to disturbances, herbivory, and weather, for example, so a comprehensive theory of plant community dynamics much include both density and frequency dependence. If densities are high enough to result in CFY in the field, however, our results suggest that density may not need to be considered. Competitive interactions among plant species will only be predictable at high density, while lower densities produce “transient dynamics,” which are dominated by stochastic factors.

Although our experiments were primarily on annual plants, our results may be most directly relevant to perennial systems, because CFY applies to biomass, and competitive interactions in perennial systems occur as some species grow at the expense of others (Adler et al., 2010). Seed production by single species does not show CFY but often declines with density at high densities, as we observed for those species that produced seeds in our experiments, so we cannot simply apply biomass results to predict reproductive output. Still, there is a high correlation between plant size and reproductive output and seed production within species, and seed production does level off at very high densities, even if it is somewhat higher at a less high density (Weiner & Freckleton, 2010), so our results are also relevant to seed production in communities with densities well above the minimum for CFY.

## CONCLUSIONS

Our results generally support the hypothesis that constant final yield applies to plant communities, perhaps even more than it applies to monocultures, and this has important implications for plant communities.

The CP of species, and therefore their contributions to total community biomass in mixtures, changes with density up to densities that produce CFY, but not at yet higher densities.

Our results demonstrate that density is an essential dimension that needs to be considered in theoretical and experimental research on plant community dynamics. Though there has been much focus on the role of frequency dependence for the coexistence of plant species, our results suggest that frequency dependence is itself density dependent at densities below CFY. The behavior of plant communities will be most consistent and, therefore, predictable at densities high enough to result in CFY.

## AUTHOR CONTRIBUTIONS

Jacob Weiner conceived the ideas behind the study. Andrea Cavalieri and Jacob Weiner designed the experiments. Andrea Cavalieri performed Experiments 1 and 2. Andrea Cavalieri, Dorothee Groß, and Alexandra Dutay performed Experiment 3. All authors provided editorial feedback on the manuscript. All authors have read and agreed to the content of the manuscript.

## CONFLICT OF INTEREST

The authors declare no conflict of interest.

## Supporting information


Appendix S1

## Data Availability

Data (Cavalieri & Weiner, 2021) are available from the University of Copenhagen Electronic Research Data Archive at https://doi.org/10.17894/UCPH.7C2FE4F5-0EB3-47F3-A88B-8877B2E2D1E4.
