# Supplementary material for: Do plant communities show constant final yield?
Source: Ecology. 2022 Aug 18;103(11):e3802. doi: 10.1002/ecy.3802 (PMC9788247; doi:10.1002/ecy.3802)
Supplement: Supplementary file 1 — Appendix S1 [file ECY-103-e3802-s001.pdf]

Supporting Information. Cavalieri, A., D. Groß, A. Dutay, and J. Weiner. Do plant communities show constant final yield? Ecology.

APPENDIX S1

TABLE S1. Dead biomass (g m^{-2}) produced by monocultures in Experiment 1. Within each column means followed by different letters indicate significant differences between means using Fishers protected LSD (alpha = 0.05). Mean ± SD (n = 3).

Density	*M. syslvestris*	*P. lanceolata*	*R. acetosa*	*L. multiflorum*	*T. repens*
1	15.41 ± 6.69 a	3.02 ± 2.33 c	2.66 ± 2.05 a	2.79 ± 1.94 c	0.56 ± 0.31 b
2	22.36 ± 7.81 a	3.92 ± 2.87 bc	3.04 ± 2.74 a	2.24 ± 0.51 c	1.66 ± 1.43 b
3	19.04 ± 5.62 a	7.59 ± 5.12 b	4.42 ± 0.90 a	6.03 ± 1.70 bc	6.92 ± 8.21 ab
4	18.70 ± 2.80 a	5.46 ± 5.47 bc	4.24 ± 1.06 a	9.68 ± 1.93 ab	7.29 ± 5.11 ab
5	20.52 ± 2.56 a	11.58 ± 4.74 a	3.16 ± 1.31 a	10.51 ± 5.02 a	9.82 ± 9.10 a
P-value	*0.3091*	*0.0050*	*0.6772*	*0.0031*	*0.1238*
F-value	*1.43*	*8.83*	*0.59*	*10.21*	*2.52*
num DF	*4*	*4*	*4*	*4*	*4*
den DF	*8*	*8*	*10*	*8*	*8*

TABLE S2. Biomass of seeds (g m^{-2}) produced in the monocultures in Experiment 1. Within each column means followed by different letters indicate significant differences between means using Fishers protected LSD (alpha = 0.05). Mean ± SD (n = 3).

Density	*M. sylvestris*	*P. lanceolata*	*R. acetosa*	*L. multiflorum*	*T. repens*
1	1.50 ± 0.98 b	8.75 ± 3.82 a	1.89 ± 2.68 a	0.84 ± 1.14 a	-
2	6.44 ± 4.92 a	6.49 ± 4.87 a	0.37 ± 0.32 a	0.35 ± 0.51 a	-
3	3.01 ± 2.29 ab	3.81 ± 1.18 ab	-	0.21 ± 0.20 a	-
4	0.43 ± 0.37 b	0.14 ± 0.24 b	-	-	-
5	0.14 ± 0.18 b	0.05 ± 0.09 b	-	-	-
P-value	*0.0334*	*0.0156*	*0.3125*	*0.3537*	-
F-value	*4.52*	*6.01*	*1.37*	*1.28*	-
num DF	*4*	*4*	*4*	*4*	-
den DF	*8*	*8*	*10*	*8*	-

TABLE S3. Biomass of seeds (g m^{-2}) produced by the five-species mixture in Experiment 1. Within each column means followed by different letters indicate significant differences between means using Fishers protected LSD (alpha = 0.05). Mean ± SD (n = 3).

Density	*M. sylvestris*	*P. lanceolata*	*R. acetosa*	*L. multiflorum*	*T. repens*
1	0.22 ± 0.10 b	1.27 ± 1.11 a	0.29 ± 0.51	-	-
2	0.98 ± 0.83 a	0.88 ± 0.64 b	-	-	-
3	0.95 ± 0.22 a	0.50 ± 0.63 b	-	-	-
4	0.77 ± 0.52 ab	-	-	-	-
5	0.06 ± 0.02 b	-	-	-	-
P-value	*0.1086*	*0.1225*	*0.4516*	-	-
F-value	*2.69*	*2.53*	*1.00*	-	-
num DF	*4*	*4*	*4*	-	-
den DF	*8*	*8*	*10*	-	-

TABLE S4. Dead biomass (g m^{-2}) produced by the five-species mixture in Experiment 1. Within each column means followed by different letters indicate significant differences between means using Fishers protected LSD (alpha = 0.05). Mean ± SD (n = 3).

Density	*M. syslvestris*	*P. lanceolata*	*R. acetosa*	*L. multiflorum*	*T. repens*
1	0.00 ± 0.00 b	0.12 ± 0.21 a	0.79 ± 0.86 a	0.00 ± 0.00 c	1.30 ± 2.14 a
2	3.50 ± 1.92 a	0.78 ± 0.56 a	0.46 ± 0.40 a	0.49 ± 0.59 bc	0.37 ± 0.39 a
3	3.80 ± 2.22 a	0.95 ± 0.53 a	0.14 ± 0.24 a	1.46 ± 0.56 ab	0.94 ± 1.37 a
4	5.16 ± 2.14 a	3.90 ± 5.69 a	0.56 ± 0.58 a	2.39 ± 0.75 a	0.00 ± 0.00 a
5	2.76 ± 1.17 ab	0.51 ± 0.61 a	0.25 ± 0.32 a	1.46 ± 0.54 ab	0.00 ± 0.00 a
P-value	*0.0408*	*0.4394*	*0.4970*	*0.0037*	*0.5691*
F-value	*3.76*	*1.03*	*0.92*	*9.65*	*0.77*
num DF	*4*	*4*	*4*	*4*	*4*
den DF	*10*	*10*	*8*	*8*	*10*

TABLE S5. Dead biomass (g m^{-2}) produced by monocultures in Experiment 2, 2017 (n = 1).

Density	*C. cyanus*	*T. repens*	*D. moldavica*	*L. multiflorum*	*P. psyllium*
1	1.10	-	-	-	-
2	5.74	4.37	-	-	-
3	6.23	-	-	-	-
4	8.50	-	-	7.32	-
5	6.13	5.88	-	9.94	-

TABLE S6. Dead biomass (g m^{-2}) produced by monocultures in Experiment 2, 2018 (n = 1).

Density	*C. cyanus*	*T. repens*	*D. moldavica*	*L. multiflorum*	*P. psyllium*
1	-	-	-	-	-
2	-	-	-	-	-
3	3.88	-	-	1.14	-
4	4.45	4.70	-	1.39	-
5	-	-	-	3.56	-

TABLE S7. Dead biomass (g m^{-2}) produced by the five-species mixture in Experiment 2, 2017. Within each column means followed by different letters indicate significant differences between means using Fishers protected LSD (alpha = 0.05). Mean ± SD (n = 3).

Density	*C. cyanus*	*T. repens*	*D. moldavica*	*L. multiflorum*	*P. psyllum*
1	-	-	-	-	-
2	0.75 ± 0.66 c	-	-	0.49 ± 0.42 c	-
3	2.13 ± 0.95 b	-	-	0.17 ± 0.16 c	-
4	2.48 ± 0.19 b	-	0.02 ± 0.03	1.04 ± 0.39 b	-
5	4.48 ± 0.81 a	-	-	2.66 ± 0.32 a	0.10 ± 0.17
P-value	*> 0.0001*		*0.4516*	*> 0.0001*	*0.4516*
F-value	*22.11*		*1.00*	*61.68*	*1.00*
num DF	*4*		*4*	*4*	*4*
den DF	*10*		*10*	*8*	*10*

TABLE S8. Dead biomass (g m^{-2}) produced by the five-species mixture in Experiment 2, 2018. Within each column means followed by different letters indicate significant differences between means using Fishers protected LSD (alpha = 0.05). Mean ± SD (n = 3).

Density	*C. cyanus*	*T. repens*	*D. moldavica*	*L. multiflorum*	*P. psyllum*
1	-	-	-	-	-
2	0.51 ± 0.44 c	-	-	0.23 ± 0.21 b	-
3	0.70 ± 0.60 bc	-	-	0.22 ± 0.21 b	-
4	3.31 ± 1.18 a	-	-	0.94 ± 0.14 a	-
5	1.96 ± 0.82 b	-	-	0.75 ± 0.07 a	-
P-value	*0.0015*			*0.0001*	
F-value	*10.23*			*25.38*	
num DF	*4*			*4*	
den DF	*10*			*8*	

TABLE S9. Dead biomass (g m^{-2}) produced by the five-species mixture in Experiment 3, 2017. Within each column means followed by different letters indicate significant differences between means using Fishers protected LSD (alpha = 0.05). Mean ± SD (n = 3).

Density	*C. cyanus*	*C. officinalis*	*D. moldavica*	*L. multiflorum*	*P. psyllium*
1	0.56 ± 0.96 b	2.91 ± 2.13 a	0.52 ± 0.91 a	0.98 ± 0.85 c	0.49 ± 0.85 a
2	4.33 ± 0.83 a	3.77 ± 1.79 a	2.00 ± 1.74 a	2.58 ± 0.82 ab	-
3	4.95 ± 0.52 a	4.28 ± 0.92 a	2.05 ± 0.99 a	2.14 ± 0.95 bc	0.50 ± 0.87 a
4	6.24 ± 1.15 a	4.14 ± 0.96 a	1.59 ± 0.11 a	2.20 ± 0.87 bc	1.52 ± 1.53 a
5	6.86 ± 2.75 a	4.03 ± 1.07 a	1.57 ± 0.09 a	3.69 ± 0.18 a	1.11 ± 0.97 a
P-value	*0.0030*	*0.7952*	*0.2683*	*0.0227*	*0.4030*
F-value	*8.48*	*0.41*	*1.58*	*4.62*	*1.11*
num DF	*4*	*4*	*4*	*4*	*4*
den DF	*10*	*10*	*8*	*10*	*10*

TABLE S10. Dead biomass (g m^{-2}) produced by the five-species mixture in Experiment 3, 2018. Within each column means followed by different letters indicate significant differences between means using Fishers protected LSD (alpha = 0.05). Mean ± SD (n = 3).

Density	*C. cyanus*	*C. officinalis*	*D. moldavica*	*L. multiflorum*	*P. psyllium*
1	0.05 ± 0.08 b	0.28 ± 0.26 c	0.08 ± 0.13 ab	0.03 ± 0.05 d	0.03 ± 0.05 a
2	0.05 ± 0.09 b	0.29 ± 0.29 c	0.07 ± 0.12 ab	0.01 ± 0.02 d	0.03 ± 0.06 a
3	0.56 ± 0.40 b	0.46 ± 0.10 bc	0.06 ± 0.10 ab	0.24 ± 0.11 c	0.04 ± 0.06 a
4	0.74 ± 0.31 ab	0.74 ± 0.08 ab	0.04 ± 0.08 b	0.78 ± 0.13 b	-
5	1.68 ± 1.11 a	0.91 ± 0.14 a	0.25 ± 0.08 a	1.25 ± 0.18 a	0.07 ± 0.06 a
P-value	*0.0249*	*0.0083*	*0.1795*	*> 0.0001*	*0.6077*
F-value	*4.48*	*6.33*	*1.94*	*70.96*	*0.70*
num DF	*4*	*4*	*4*	*4*	*4*
den DF	*10*	*10*	*10*	*8*	*10*

TABLE S11. Percentage survival {[(number of plants harvested)/(number of plants emerged)] x 100} in the five-species mixture in Experiment 3 in 2017 and 2018. Within each column means followed by different letters indicate significant differences between means using Fishers protected LSD (alpha = 0.05). Mean ± SD (n = 3). *: mean number of plants harvested was greater than mean number counted after emergence due to delayed emergence

	Density	*C. officinalis*	*C. cyanus*	*D. moldavica*	*L. multiflorum*	*P. psyllium*
2017						
	1	100 ± 0 a	100 ± 0 a	100 ± 0 a	100 ± 0 a	100 ± 0 a
	2	100 ± 0 a	*	100 ± 0 a	*	*
	3	*	89 ± 17 a	97 ± 19 a	*	80 ± 44 a
	4	83 ± 12 b	92 ± 25 a	91 ± 20 a	71 ± 21 b	90 ± 15 a
	5	*	93 ± 8 a	80 ± 4 a	73 ± 15 b	76 ± 9 a
2018						
	1	100 ± 0 a	100 ± 0 a	100 ± 0 a	*	83 ± 29 a
	2	100 ± 20 a	100 ± 0 a	100 ± 0 a	*	93 ± 12 a
	3	99 ± 23 a	*	64 ± 43 a	*	*
	4	97 ± 15 a	*	88 ± 16 a	*	99 ± 16 a
	5	58 ± 9 b	*	63 ± 9 a	92 ± 21	55 ± 14 b
